# Supplementary material for: Reactive oxygen species-dependent Toll/NF-κB activation in the Drosophila hematopoietic niche confers resistance to wasp parasitism
Source: eLife. 2017 Nov 1;6:e25496. doi: 10.7554/eLife.25496 (PMC5681226; doi:10.7554/eLife.25496)
Supplement: Transparent reporting form [file elife-25496-transrepform.pdf]

eLIFE
1st Floor
24 Hills Road
Cambridge CB2 1JP, UK
P 01223 855340
W elifesciences.org
T @elife

eLife's transparent reporting form

We encourage authors to provide detailed information *within their submission* to facilitate the interpretation and replication of experiments. If you have any questions, please contact us: editorial@elifesciences.org.

Sample-size estimation

- You should state whether an appropriate sample size was computed when the study was being designed
- You should state the statistical method of sample size computation and any required assumptions
- If no explicit power analysis was used, you should describe how you decided what sample (replicate) size (number) to use

Please outline where this information can be found within the submission (e.g., page numbers or figure legends), or explain why this information doesn't apply to your submission:

Quantification of lymph gland disruption: Fig 1 C,F,G; Fig 2J; Fig 4A; Fig 5A-C; Fig 6L,M; Fig 7C,D,G; Fig 1-S2, Fig 4-S1, Fig 5-S1. Numbers of lymph glands analyzed for each genotype are indicated on the vertical bars of graphs.
Quantification of wasp egg hatching: Fig 2C,K; Fig 4D. Fig 6N,O; Fig 2-S1; Fig 5-S1. Numbers of larvae analyzed for each genotype are indicated on the vertical bars of graphs.
Quantification of staining mean intensity (D4-lacZ or GstD-lacZ) in PSC cells: Fig 3E,H; Fig 5F; Fig 6C,D,G,H,K. For each genotype, the number of PSC analyzed is plotted on graphs.
DIC and confocal images: Representative images of at least 20 anterior lobes analyzed per experiment (see Materials and methods).

Replicates

- You should report how often each experiment was performed
- You should include a definition of biological versus technical replication
- The data obtained should be provided and sufficient information should be provided to indicate the number of independent biological and/or technical replicates
- If you encountered any outliers, you should describe how these were handled
- Criteria for exclusion/inclusion of data should be clearly stated
- High-throughput sequence data should be uploaded before submission, with a private link for reviewers provided (these are available from both GEO and ArrayExpress)

Please outline where this information can be found within the submission (e.g., page numbers or figure legends), or explain why this information doesn't apply to your submission:

Quantification of lymph gland disruption: Fig 1 C,F,G; Fig 2J; Fig 4A; Fig 5A-C; Fig 6L,M; Fig 7C,D,G; Fig 1-S2, Fig 4-S1, Fig 5-S1. In all experiments, genotypes were analyzed in parallel. At least 3 independent biological replicates were performed and quantified. The mean of at least 3 experiments is presented (see Materials and methods).
Quantification of wasp egg hatching: Fig 2C,K; Fig 4D. Fig 6N,O; Fig 2-S1; Fig 5-S1. In all experiments, genotypes were analyzed in parallel. At least 3 independent biological replicates were performed and quantified. The mean of at least 3 experiments is presented (see Materials and methods).
Quantification of staining mean intensity (D4-lacZ or GstD-lacZ) in PSC cells: Fig 3E,H; Fig 5F; Fig 6C,D,G,H,K. In all experiments, genotypes were analyzed in parallel. At least 3 independent biological replicates were performed and quantified. One quantification is shown (see Materials and methods).
DIC and confocal images: 3 independent biological replicates were analyzed and one representative image is shown (see Materials and methods).

Statistical reporting

- Statistical analysis methods should be described and justified
- Raw data should be presented in figures whenever informative to do so (typically when N per group is less than 10)
- For each experiment, you should identify the statistical tests used, exact values of N, definitions of center, methods of multiple test correction, and dispersion and precision measures (e.g., mean, median, SD, SEM, confidence intervals; and, for the major substantive results, a measure of effect size (e.g., Pearson's r, Cohen's d)
- Report exact p-values wherever possible alongside the summary statistics and 95% confidence intervals. These should be reported for all key questions and not only when the p-value is less than 0.05.

Please outline where this information can be found within the submission (e.g., page numbers or figure legends), or explain why this information doesn't apply to your submission:

Quantification of lymph gland disruption: Fig 1 C,F,G; Fig 2J; Fig 4A; Fig 5A-C; Fig 6L,M; Fig 7C,D,G; Fig 1-S2, Fig 4-S1, Fig 5-S1. Error bars correspond to SEM, P-values were calculated using a Pearson's Chi-squared test. Both are indicated on graphs and in the legend of Figure 1.
Quantification of wasp egg hatching: Fig 2C,K; Fig 4D. Fig 6N,O; Fig 2-S1; Fig 5-S1. Error bars correspond to SEM, P-values were calculated using a Pearson's Chi-squared test. Both are indicated on graphs and in the legend of Figure 2.
Quantification of staining mean intensity (D4-lacZ or GstD-lacZ) in PSC cells: Fig 3E,H; Fig 5F; Fig 6C,D,G,H,K. Statistical analysis t-test (Mann-Whitney nonparametric test) were performed. For Fig 3E, unpaired t-test with Welch's correction is shown. P-values and SDs are given on graphs.

(For large datasets, or papers with a very large number of statistical tests, you may upload a single table file with tests, Ns, etc., with reference to page numbers in the manuscript.)

Additional data files ("source data")

- We encourage you to upload relevant additional data files, such as numerical data that are represented as a graph in a figure, or as a summary table
- Where provided, these should be in the most useful format, and they can be uploaded as "Source data" files linked to a main figure or table
- Include model definition files including the full list of parameters used
- Include code used for data analysis (e.g., R, MatLab)
- Avoid stating that data files are "available upon request"

Please indicate the figures or tables for which source data files have been provided:

Figure 1-Source data 1. Lymph gland disruption quantification.
Figure 2-Source data 1 and 3. Wasp egg hatching quantifications.
Figure 2-Source data 2. Lymph gland disruption quantification.
Figure 3-Source data 1 and 2. D4-lacZ staining quantifications.
Figure 3-Source data 3. Lymph gland disruption quantification.
Figure 3-Source data 4. Wasp egg hatching quantification.
Figure 4-Source data 1, 2 and 3. Lymph gland disruption quantifications.
Figure 5-Source data 1, 2. gstD-lacZ staining quantifications.
Figure 5-Source data 3. D4-lacZ staining quantification.
Figure 5-Source data 4 and 5. Lymph gland disruption quantifications.
Figure 6-Source data 1, 2 and 3. Lymph gland disruption quantifications.
